# Dual Targeting of Pim and PI3 Kinases in Mature T‐Cell Lymphoma

**DOI:** 10.1111/ejh.14420

**Published:** 2025-03-31

**Authors:** M. Lohrberg, M. Heber, L. Ries, K. Markus, N. Ksionsko, N. Schmidt, G. Nothnick, L. Thielking, M. O'Neill, S. Martínéz‐González, C. Blanco‐Aparicio, J. Pastor, D. Cunningham, R. Koch

**Affiliations:** ^1^ Haematology and Medical Oncology University Medical Center Göttingen Göttingen Germany; ^2^ Inflection Biosciences Ltd. Dublin Ireland; ^3^ Centro Nacional de Investigaciones Oncológicas Madrid Spain

**Keywords:** AZD1208, IBL‐202, lymphoma, PI3K, Pim kinase, T‐cell lymphoma

## Abstract

Provirus Integration site for Moloney leukemia virus (Pim) family members are well‐known oncogenes, with an expression that is restricted to few cell types including hematopoietic cells in adult organisms, making it a promising target for lymphoma treatment. Indeed, previous studies in mature T‐cell lymphoma (mTCL) cells revealed frequent upregulation of Pim expression. Nevertheless, inhibition of Pim kinases showed only minor effects on the viability of mTCL cells so far. Thus, we here addressed cellular responses to therapeutic inhibition of Pim kinases and identified a PI3K/Akt‐driven activation of mTOR as a significant escape mechanism mitigating the anti‐lymphoma effects of Pim inhibition. Indeed, dual inhibition of Pim and PI3 kinases showed synergistic anti‐lymphoma effects in vitro through downregulation of mTOR‐induced protein translation and mitigation of BCL‐xL‐mediated anti‐apoptotic mechanisms. Based on this finding, we next explored the therapeutic potential of the dual Pim/PI3K inhibitor IBL‐202 in mTCL cell lines. Strikingly, IBL‐202 strongly induced cell‐cycle‐dependent cell death in cell lines of different mTCL subtypes. Together, our study provides mechanistic evidence supporting a therapeutic strategy of dual Pim‐ and PI3‐kinase inhibition in mature T‐cell lymphoma.

## 1 Introduction

Mature T‐cell lymphomas (mTCL) are a heterogeneous and largely aggressive group of lymphoid malignancies. These tumors develop from post‐thymic T‐cells of different stages and show a diverse histopathologic and clinical picture, with 33 different entities classified according to WHO [1]. Employing currently established therapeutics, the prognosis for most subtypes of mTCL remains very poor. Still, molecular studies on mTCL recently provided significant insight into the molecular pathogenesis of these diseases and identified molecular alterations that might serve as a basis for targeted therapeutic strategies.

Indeed, molecular studies identified signaling pathways that are activated across different subtypes, including the JAK/STAT‐, PI3K/Akt‐, NOTCH‐, and RHOA‐pathways [2]. Although these pathways are known as oncogenic drivers, therapeutic inhibition is challenged by their relevance in physiological cellular processes, leading to severe side effects. A potential therapeutic target that is more restricted to malignant T‐cells, though, could be the protein family of *P*rovirus *I*ntegration site for *M*oloney leukemia virus (Pim) kinases. Their expression pattern is broad during embryonic development but is mostly restricted to hematopoietic cells and the gastrointestinal and urinary tracts in adult human organisms. Although the three family members (Pim1, ‐2, ‐3) are encoded on different chromosomes and only show amino acid homologies of around 50%, they are most probably compensatory to each other. Notably, Pim kinase expression is often upregulated in hematologic cancer cells (reviewed in: [3]).

Pim kinases are constitutively active and in cancer cells activation is enhanced by different positive feedback loops, including JAK/STAT, PI3K/Akt, and NF‐κB pathways. Pim kinases are involved in various cellular mechanisms, including cell cycle regulation, transcriptional regulation, regulation of mRNA translation, and apoptosis (reviewed in: [4]).

Importantly, recent analyses identified an enhanced activation of Pim kinases in mTCL samples compared to reactive T‐cells. Notably, siRNA‐mediated knockdown of Pim kinases only led to apoptosis in about 20%–30% of mTCL cells, which can potentially be explained by the compensatory function of the three Pim kinases. Inhibition of all Pim kinases using the pharmacologic pan‐Pim inhibitor ETP‐39010 significantly reduced cell viability in mTCL cell lines [5]. Indeed, further preclinical evidence supports a potential therapeutic relevance of pan‐PIM inhibitors in hematologic malignancies. Different studies could show selective activity of the pan‐PIM inhibitor AZD1208 in hematologic malignancies, such as Bellon and co‐workers who analyzed the effect of AZD1208 on Adult T‐cell leukemia (ATL) cells in vitro and showed induction of cell death in ATL cells, but not peripheral blood mononuclear cells (PBMCs) [6]. Moreover, xenograft studies using Acute lymphocytic leukemia (ALL) cell lines and T‐cell lymphoblastic lymphoma (T‐LBL) patient cells were performed in mice, showing that AZD1208 treatment led to significantly decreased spleen/tumor weight and volume, as well as delayed tumor development [6, 7]. Still, clinical data on the safety and efficacy of Pim kinase inhibitors for hematologic malignancies is sparse. So far, the pan‐Pim inhibitor Pim447 was tested in a phase I clinical trial in patients with multiple myeloma (MM) and showed a clinical benefit rate of 25.3% and a disease control rate of 72.2%. Notably, Pim447 did not induce detectable pro‐apoptotic but rather cytostatic effects [8]. In a phase I trial of AZD1208 in acute myeloid leukemia (AML) patients, AZD1208 led to disease stabilization in some cases but did not induce clinical remissions [9]. Thus, despite promising results of preclinical studies highlighting a potential therapeutic relevance of Pim kinases in hematologic malignancies, the clinical benefit is disappointing so far, which raises questions about potential mechanisms mitigating the effects of therapeutic Pim inhibition. This study aims to understand the cellular effects of pharmacological Pim inhibition in mature T‐cell lymphoma (mTCL), with specific focus on cell‐intrinsic pathways and potential combination strategies to overcome therapy resistance.

## 2 Material and Methods

### 2.1 Cell Culture

The mTCL cell lines and culture conditions are specified in Table S1.

### 2.2 Western Blot Analysis

For semi‐quantitative determination of protein expression and phosphorylation status, western blot analysis was performed according to standard protocols (for details see Data S1). Quantification was done using the software FIJI [10].

### 2.3 AlamarBlue Cell Viability Assay

To determine the toxicity of the different inhibitors (AZD1208, AstraZeneca, Cambridge, GBR; Copanlisib, trade name Aliqopa, BAYER, Leverkusen, GER; IBL‐202, Inflection Biosciences, Dublin, IRL) on the different mTCL cell lines, alamarBlue Cell Viability Reagent was used and manufacturer's instructions were followed (Cat. No. DAL 1025, Thermo Fisher). For details, see Data S1.

The same protocol was followed to determine synergistic effects of AZD1208 and copanlisib. The expected drug combination response was calculated based on a zero inhibitory potential (ZIP) or highest single agent (HSA) reference model using the software SynergyFinder 2.0 (https://synergyfinder.fimm.fi, [11]). Deviations between observed and expected responses with positive and negative values denote synergy and antagonism, respectively.

### 2.4 Calculation of GR_max_ , GR_50_ , and IC_50_

To determine the correlations between the genotype of the different mTCL cell lines and the drug sensitivity, the metrics of tested compounds were determined using the GR Calculator software tool (http://www.grcalculator.org; [12]). In particular, the half‐maximal inhibitory concentration (IC_50_) as well as the growth rate inhibition (GR) were calculated. GR contains two different values, namely those are GR_50_ (drug potency) and GR_max_ (drug efficiency).

### 2.5 FACS‐Based Propidium Iodide Cytotoxicity Assay

To quantify cell death in response to treatment, time course experiments were performed. Therefore, mTCL cells were seeded into 96‐well microplates in normal culture media (10 000 cells per well). Subsequently, the test compound was added to the cell suspension as specified later. After 24, 48, or 72 h, the amount of dead cells was measured by staining with propidium iodide (PI, 1:4, Sigma‐Aldrich #537059) and flow cytometry‐based analysis using a BD LSRFortessa X‐20.

### 2.6 Dynamic BH3 Profiling

This method was used to investigate the functional effects of AZD1208 and copanlisib on the regulation of apoptosis. The method of intracellular BH3 profiling (iBH3) measures the release of mitochondrial cytochrome C in response to a library of synthetic peptides that specifically manipulate components of the mitochondrial apoptosis pathway. To do so, cells were treated with Pim‐ or PI3K‐specific inhibitors, exposed to BH3 peptides, stained with a fluorophore‐labeled anti‐CytoC antibody, and analyzed via flow cytometry (BD LSRFortessa X‐20), as published previously [13]. This method has been invented and published by the Letai Lab at Dana Farber Cancer Research Institute ([14]; https://patents.google.com/patent/US10393733B2/en). Permission for usage has been obtained. For details, see Data S1.

### 2.7 Kinomics

Kinase assays for PI3K, mTOR, and Pim were performed by CNIO laboratories. The experimental procedure is described as Data S1.

KINOMEScan was performed by Eurofins LeadHunter Discovery Services to characterize the interaction between IBL‐202 and 455 kinases. The method has been described elsewhere [15] and generates thermodynamic interaction affinities at an IBL‐202 concentration of 1000 nM. Results were mapped using the TREE*spot* Software Tool (http://www.eurofinsdiscovery.com/StudyManagement/TREEspot/). The IC_50_ of IBL‐202 acting on TTK was determined by Eurofins KinaseProfiler. Therefore, an enzymatic radiometric assay was applied.

### 2.8 Cell Cycle Analysis

For cell cycle analysis, the same pre‐staining procedure was used as described for the PI‐based cytotoxicity assay. After a 72 h incubation time, 10 μL culture medium with 1:4 Hoechst 33342 was added and incubated for 45 min in the dark. Then Hoechst staining was analyzed by FACS (BD LSRFortessa X‐20) and flow cytometry results were analyzed using FlowJo v10.8 Software (BD Life Sciences).

## 3 Results

### 3.1 Expression Profiles of Pim Kinases Differ Among mTCL Cell Lines

Initially, previously published RNA and whole exome sequencing (WES) data was re‐analyzed to screen 17 established mTCL lines for mRNA expression levels and copy number variations (CNVs) of Pim kinases [12]. Data were mapped using the open‐source platform Morpheus (Figure S1A,B) (Morpheus, https://software.broadinstitute.org/morpheus). Chosen cell lines represent different subgroups of mTCL, in particular ALK+ anaplastic large cell lymphoma (ALCL), ALK− ALCL, PTCL not otherwise specified (PTCL‐NOS), hepatosplenic T‐cell lymphoma (HSTCL), cutaneous T‐cell lymphoma (CTCL) and natural‐killer/T‐cell lymphoma (NKTCL).

Pim mRNA was detectable in all lines with varying expression levels. The highest expression could be detected in DERL2 (Pim1), FEPD, and SMZ1 (Pim2), and KARPAS‐299 (Pim3) (Figure S1). CNVs were mainly detectable at the Pim2 gene locus, with deletions in 7 out of 15 mTCL cell lines of different subtypes. For Pim1 and Pim3, CNVs were less common. Gene amplifications were restricted to ALCL ALK− subtype (Pim1: DL40; Pim3: MAC2a) (Figure S1). However, CNVs were not reflected by mRNA expression profiles. Western blot analysis showed varying protein levels for all Pim kinase isoforms, with mostly minor up‐ or down‐regulations across subtypes (Figure S1).

### 3.2 Pim Kinase Inhibitor AZD1208 Inhibits Proliferation of mTCL Cells, With Only Minor Cytotoxicity

**FIGURE 1:**
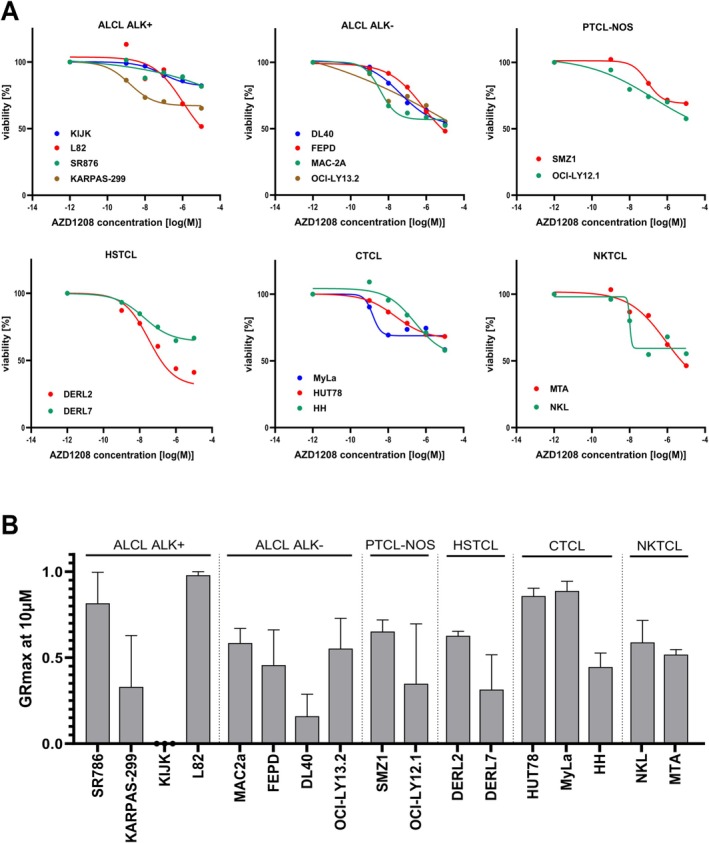
Impact of Pim kinase inhibition on cell viability. Alamar Blue assay was performed after 72 h of AZD1208 treatment to measure the impact of Pim kinase inhibiton on cell viability. (A) Different concentrations of AZD1208 were tested and viability [%] was plotted against log(M) of the AZD1208 concentration used. Lines represent non‐linear regression for the obtained data for each line. Results from at least three independent experiments are shown. (B) Obtained data after 72 h treatment with 10 μM AZD1208 was used to calculate GR_max_ values. Mean and SEM are depicted.

Using the AlamarBlue assay, viability of cells treated with ascending concentrations AZD1208 (10 nM to 10 μM) was measured and compared to DMSO‐treated controls. Normalized values were plotted via non‐linear regression and IC_50_s were determined (Table S2, Figure 1A). The calculated IC_50_s were in a range between 1.5 nM (KARPAS‐299 and MyLa) and 1.1 μM (L82). In most of the tested cell lines, cells' reaction was weak or nearly not detectable. Therefore, some IC_50_s could not be calculated and are indicated as ‘not determined’ (n.d.). The public available GR calculator tool was used for characterization of the response to AZD1208. Notably, GR_max_ values were consistently between 0 and +1 for all tested cell lines, indicating that inhibition of Pim kinases induces mild cytostatic but no cytotoxic effects in mTCL cells (Figure 1B) [12].

### 3.3 PI3K Inhibitor Copanlisib and Pan‐Pim Inhibitor AZD1208 Show Synergistic Anti‐Lymphoma Effects

One of the main downstream targets of Pim kinases is the mTOR pathway, which is also a key effector of the PI3K/Akt pathway that is known to be highly active in mTCL cells [16]. Considering the relevance of PI3K/Akt signaling in mTCL, we hypothesized an impact of the PI3K/Akt signaling pathway on Pim‐mediated mTOR regulation, potentially mitigating the effect of Pim inhibition.

**FIGURE 2:**
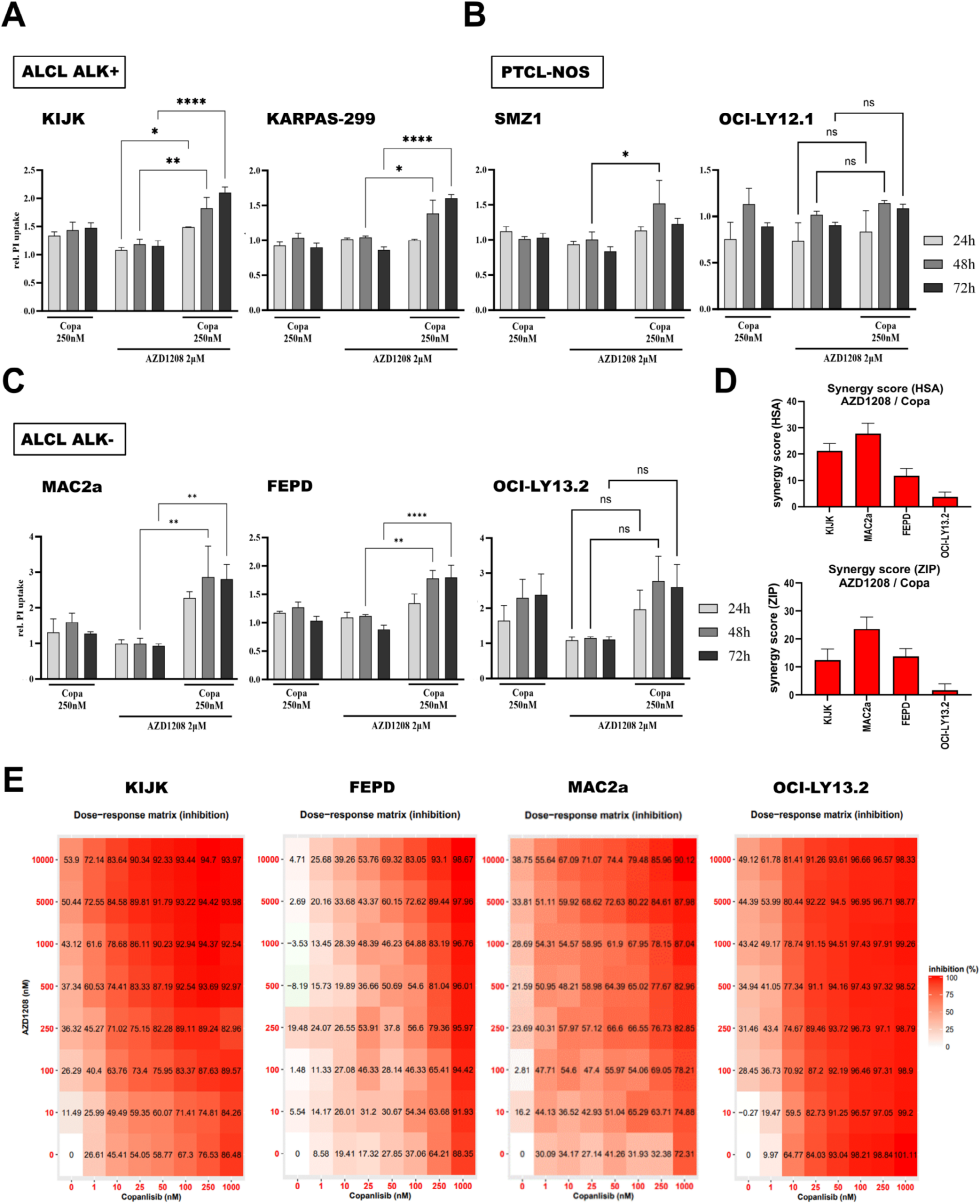
Dual inhibition of Pim kinases and PI3K. For quantification of cytotoxicity, propidium iodide (PI) uptake was measured by FACS analysis. (A–C) show relative PI uptake in mTCL cells treated with 250 nM copanlisib, 2 μM AZD1208 or combination of both after 24, 48, or 72 h. PI‐uptake after treatment was normalized to DMSO‐treated control samples (PI of DMSO group was set as +1). Results of three independent experiments are shown as mean ± SEM. Two‐way ANOVA was performed with α = 0.05 and *p < 0.05, **p < 0.01, and ***p < 0.001. Obtained data for KIJK, FEPD, MAC2a, and OCI‐LY13.2 was used to calculate synergy scores using SynergyFinder 2.0 software tool. Results are depicted either as bar diagrams (D, HSA = highest single agent, ZIP = zero interaction potency) or dose–response matrix (E).

To test this hypothesis, we treated a panel of 8 mTCL lines from different subtypes with AZD1208 in combination with the PI3K inhibitor copanlisib. FACS‐based analysis of the uptake of PI was performed after treatment with copanlisib, AZD1208, or combination and normalized to DMSO‐treated controls. Again, mono‐treatment with 2 μM AZD1208 did not induce cytotoxicity, leading to PI uptake that was comparable to control samples. PI3K inhibitor copanlisib (250 nM) induced cytotoxicity in OCI‐LY13.2 cells (Figure 2C) but did not induce PI uptake in the other seven tested samples. However, combi‐treatment with 250 nM copanlisib and 2 μM AZD1208 led to significant increase of the PI‐positive subpopulation, indicating enhanced cell death (Figure 2A–C). Indeed, further analyses using the open source software tool SynergyFinder 2.0 (https://synergyfinder.fimm.fi; [11]) calculating the “highest single agent” (HSA) or the “zero interaction potency” (ZIP) models [11] revealed synergy scores of above +10, indicating a synergistic effect of the tested drug combination AZD1208+copanlisib for 3 of 4 tested cell lines. Only synergy scores for the PTEN‐deficient cell line OCI‐LY13.2 were between 0 and +10, indicating an additive effect of the drug combination (Figure 2D). Visualization of the cells' response to the drug combination is achieved by the adjacent dose–response matrix (Figure 2E).

### 3.4 Pathway Analysis Unravels the Key Drivers of AZD1208/Copanlisib Synergy

Two main downstream targets that can be found in both the Pim‐ and PI3K‐signaling pathways are, on the one hand, mTOR and, in particular, the mTOR‐complex 1 (mTORC1) and, on the other hand, the anti‐apoptotic protein BAD.

**FIGURE 3:**
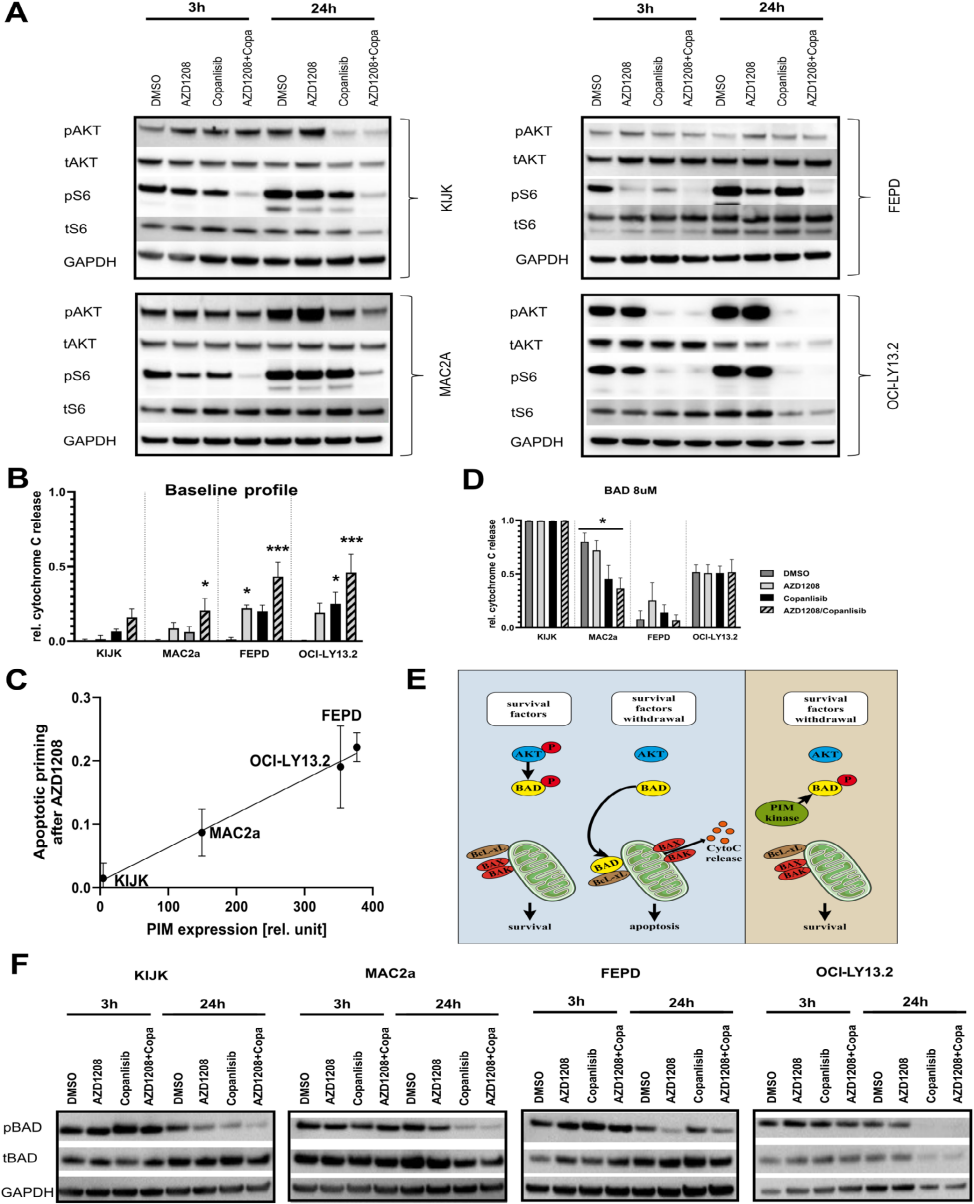
Pathway analysis to identify mechanisms of synergy. Western blot analysis of phosphorylated Akt (pAkt), total Akt (tAkt), phosphorylated S6 (pS6), total S6 (tS6), as well as of the house‐keeping gene GAPDH was performed on KIJK, MAC2a, FEPD and OCI‐LY13.2 cells. Pre‐treatment was performed for 3 or 24 h with 2 μM AZD1208, 250 nM copanlisib or combination of both. Control cells were treated with DMSO only. All western blots were performed using protein lysates of three independent experiments and representative membranes are depicted in this figure (A). Dynamic BH3 profiling was performed testing changes in Bcl‐2 dependency of KIJK, MAC2a, FEPD, and OCI‐LY13.2 cells after 72 h pre‐treatment with 2 μM AZD1208, 250 nM copanlisib or combination of both. Control cells were treated with DMSO only. (B) The relative cytochrome C release after treatment, but without further addition of BH3 peptides (so‐called baseline profile). Values were normalized to DMSO treated controls (DMSO group was set as 0). (C) The correlation of baseline cytochrome C release after AZD1208 pre‐treatment and the expression of the Pim kinases that was calculated out of western blot analysis shown in Figure S1. (D) Relative cytochrome C release in the presence of 8 μM BAD peptide. Results of three independently performed experiments are depicted as mean ± SEM. One‐way ANOVA was performed with α = 0.05 and *p < 0.05, **p < 0.01, and ***p < 0.001. A schematic view of the underlying pathway is shown in (E). (F) Western blot analysis of phosphorylated BAD (pBAD), total BAD (tBAD) as well as of the house‐keeping gene GAPDH. Pre‐treatment was performed according to (A).

First, we focused on mTORC1 and performed western blot analysis of KIJK, MAC2a, FEPD, and OCI‐LY13.2 cells treated for either 3 or 24 h with AZD1208, copanlisib, or combination. We analyzed the amount of phosphorylated Akt (pAkt) compared to DMSO‐treated control cells. Akt is acting upstream of mTOR and is not involved in the Pim kinase pathway. As expected, we could detect decreasing amounts of pAkt under copanlisib treatment in 3 out of 4 lines (except FEPD). Notably, AZD1208 mono treatment induced increased phosphorylation of Akt in all but OCI‐LY13.2 cells, suggesting a feedback loop that increases the PI3K‐pathway activity to compensate for the Pim kinase inhibition. Combination treatment was able to decrease the pAkt levels in all cells after 24 h (Figure 3A; quantification Figure S2). The amount of total Akt was widely unchanged; only OCI‐LY13.2 cells showed decreased tAkt levels after 24 h copanlisib or combination treatment (Figure 3A; quantification Figure S2).

Next, we performed western blot analysis of phosphorylated S6 (pS6) and used this as an indicator for mTOR activity. The pS6 levels only slightly decreased in response to mono treatment with varying results throughout the different lines. Moreover, treatment with AZD1208 for 3 h induced a reduction of S6 phosphorylation levels in 3 of 4 tested cell lines, but phosphorylation returned to control levels within 24 h. Only the combination treatment induced a stable decrease in S6 phosphorylation in all four tested lines. Comparable to tAkt, levels of total S6 were only decreased after 24 h copanlisib or combination treatment in OCI‐LY13.2 cells (Figure 3A; quantification Figure S2).

Beyond these effects on PI3K/mTOR‐pathway components, we further studied the functional impact of Pim inhibition on the regulation of mitochondrial apoptosis, using dynamic BH3 profiling [17]. Notably, AZD1208 mono‐treatment induces cytochrome C release that correlates with the expression levels of the three Pim kinases that have been measured by western blot analysis (compare Figure S1) (Figure 3B,C). Still, treatment with copanlisib was also able to induce apoptotic priming, and the combination of both showed a significantly increased apoptotic priming when compared to mono treatment (Figure 3B). Previous studies could show that Pim kinases mainly act on the Bcl2 family member BAD to interfere with BCL‐xL‐mediated apoptotic signaling [18]. BH3 profiling results showed that the addition of 8 μM of synthetic BAD peptide can induce cytochrome C release in all four tested lines. In KIJK, FEPD, and OCI‐LY13.2 cells, pre‐treatment with AZD1208, copanlisib, or the combination did not significantly alter the BAD‐induced apoptotic priming. Still, MAC2a pre‐treated cells were significantly less sensitive to the BAD peptide (Figure 3D,E). Western blot analysis revealed no changes in BAD phosphorylation after 3 h of treatment, whereas levels of phosphorylated BAD (pBAD) were very consistently decreased after 24 h. Mono‐treatment led to a diverse picture, with decreased pBAD levels after 24 h of AZD1208 in KIJK and FEPD but decreased pBAD levels after 24 h of copanlisib in KIJK, MAC2a, and OCI‐LY13.2. The combination of both was able to block BAD phosphorylation in all tested cell lines (Figure 3F; quantification Figure S2). Still, regulation of mitochondrial apoptosis is dependent on different pro‐ or anti‐apoptotic factors. Exposition of treated mTCL cells to a panel of 6 BH3 peptides targeting BCL‐2, BCL‐xL, MCL‐1, or BLF‐1 showed heterogeneous responses, but an overall tendency towards increased apoptotic priming after 72 h of AZD1208+copanlisib. Moreover, AZD1208+copanlisib treatment increased the sensitivity towards apoptotic priming via chemotherapeutic agents targeting those pathways (Figure S3).

**FIGURE 4:**
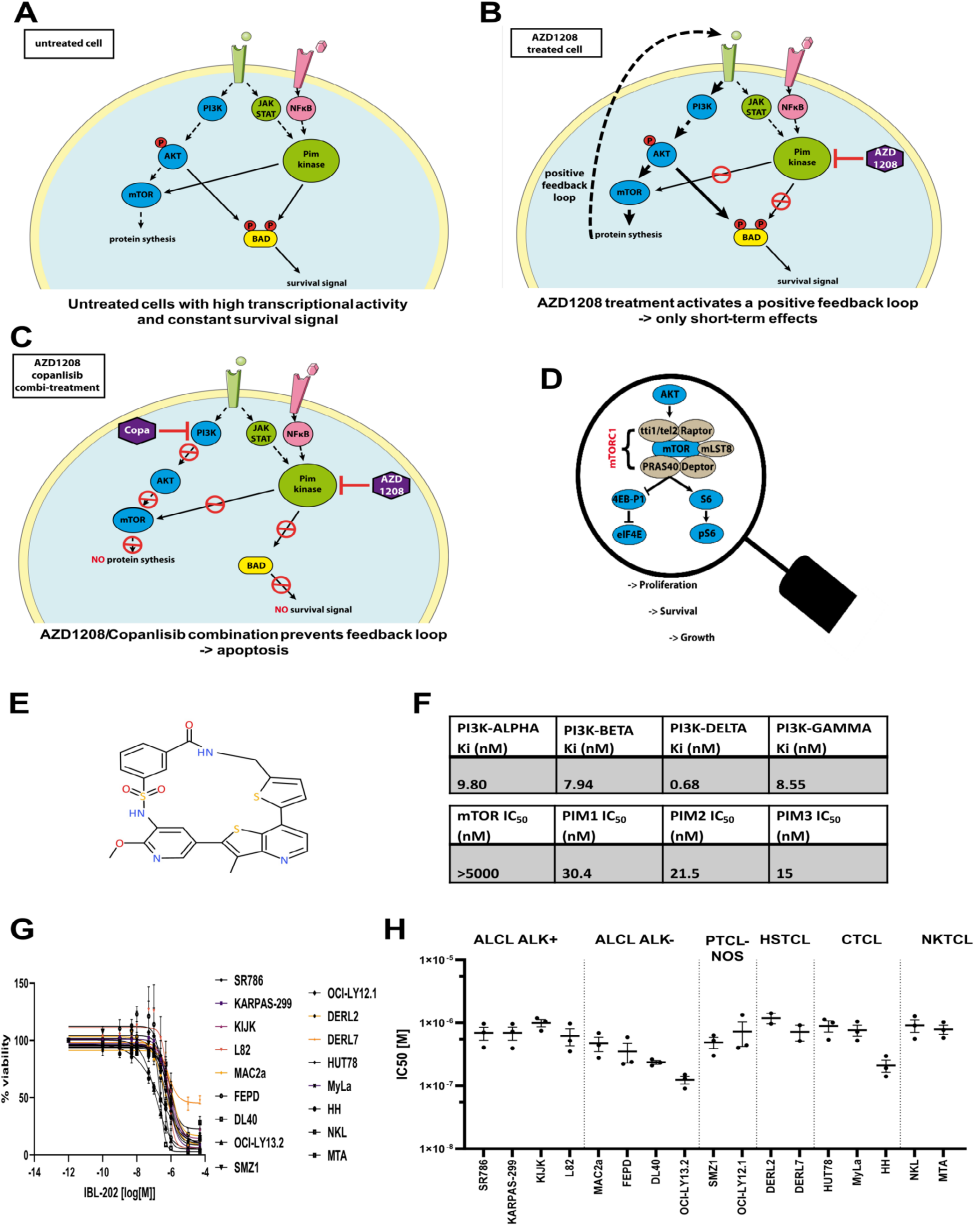
Graphical summary and introduction of the dual Pim/PI3K inhibitor IBL‐202. The hypothesized intracellular pathways acting in mTCL cells that are untreated (A), AZD1208‐treated (B) or combi‐treated (C) are depicted. The proposed positive feedback loop that prevents AZD1208‐treated cells from cell death is depicted by a dotted line (B). (D) is a more detailed view on the AKT‐activated mTOR complex 1 (mTORC1) and its downstream actors regulating cellular proliferation, survival and growth. (E) is the structural formula of IBL‐202, a dual Pim‐ and PI3K inhibitor as verified by the kinomics data listed in (F). Alamar Blue assay was performed after 72 h treatment with 1 μM IBL‐202 to measure the impact on cell viability. (G) Different concentrations of IBL‐202 were tested and viability [%] was plotted against log(M). Lines represent non‐linear regression for the obtained data for each line. Results from at least three independent experiments are shown. The half‐inhibitory concentration (IC_50_) was calculated for 17 lines and results are depicted in (H).

The proposed mode of action summarizing the observed effects is schematically depicted in Figure 4A–D.

### 3.5 Dual Pim/PI3K Inhibition Is Achieved by IBL‐202

Based on our findings, we next explored the effects of the novel inhibitor IBL‐202 [19] (Figure 4E). Supporting Information on the synthesis protocol can be found as Data S1. Kinase assays revealed strong inhibitory function of IBL‐202 on all four tested PI3K isoforms (PI3Kα, β, γ, δ) and Pim1, 2, 3, whereas mTOR was not affected (Figure 4F).

**FIGURE 5:**
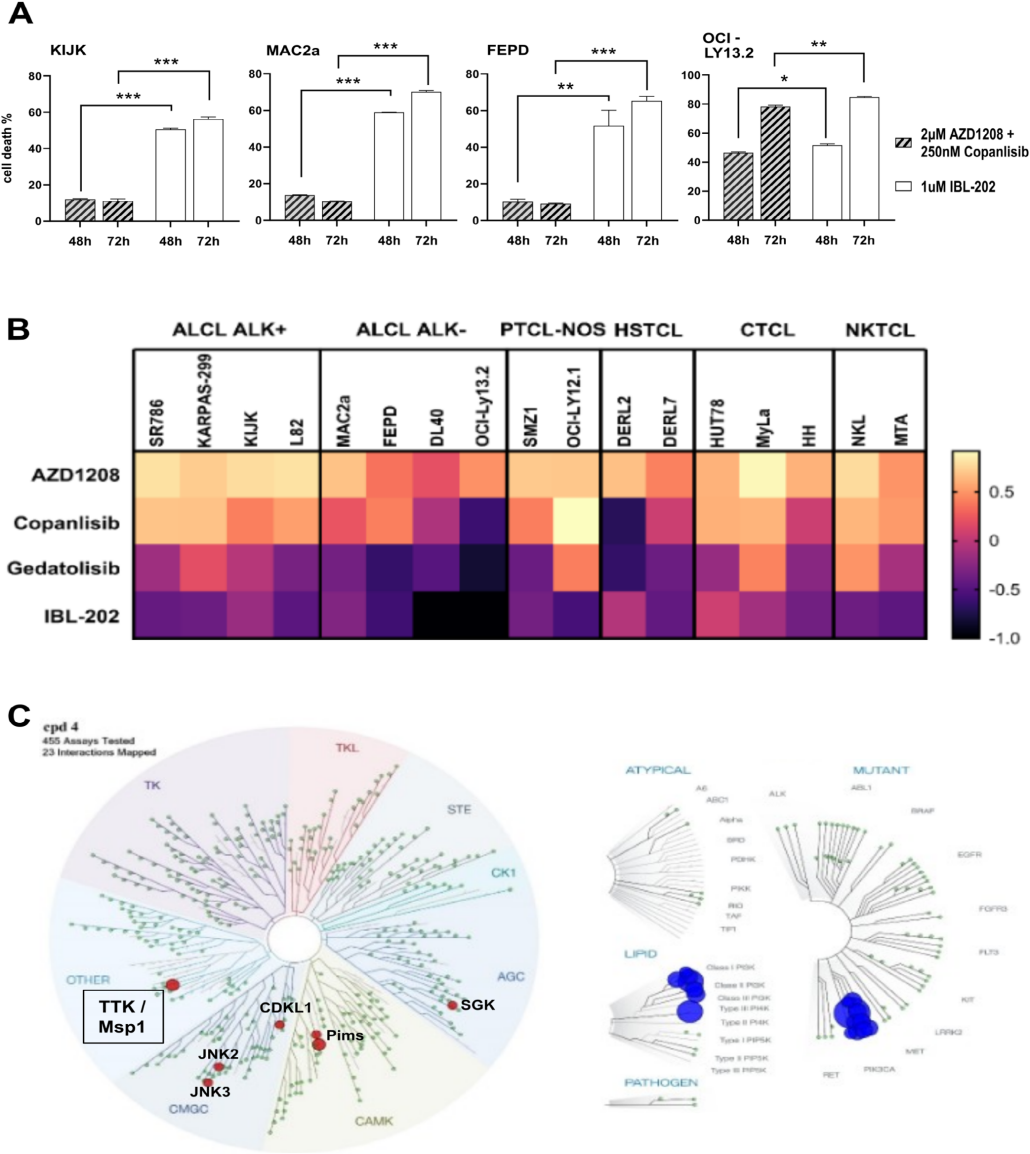
Further analysis of IBL‐202. Alamar Blue measurements were used to calculate GR_max_ values for the 17 mTCL lines after treatment with 10 μM AZD1208, copanlisib, gedatolisib (dual PI3K/mTOR inhibitor) and IBL‐202. Results are shown as a heat map (A). (B) to further analyze the cytotoxic effect of IBL‐202, PI assay was performed using KIJK, MAC2a, FEPD and OCI‐LY13.2 cells. Cells were treated either with 2 μM AZD1208+250 nM copanlisib (striped bars) or with 1 μM IBL‐202 (white bars) for 48 or 72 h. Cell death was quantified by FACS analysis of PI uptake. Results from three independently performed experiments are shown as mean ± SEM. Two‐way ANOVA was performed with α = 0.05 and *p < 0.05, **p < 0.01, and ***p < 0.001. (C) kinomics of IBL‐202 were analyzed using KINOMEScan assay and interactions are shown as a KinomeTree. Analyzed kinases are depicted as circles and red or blue circles depict interactions of IBL‐202 with this kinase. KinaseProfiler assay was used to determine the IC50s of chosen kinases. The table (D) summarizes the IC50s of the tested compounds copanlisib, AZD1208 and the mean of two batches IBL‐202.

AlamarBlue assay was performed to determine the effect of IBL‐202 on the viability and proliferation of mTCL cells. IBL‐202 showed cytotoxic effects on all 17 tested mTCL lines, as indicated in Figure 4G. The calculated IC_50_s are ranging between 124 nM (OCI‐LY13.2) and 1 μM (DERL2) (Figure 4H). To compare the cytotoxicity of IBL‐202 to AZD1208+copanlisib combi‐treatment, we treated 4 of the mTCL lines (KIJK, MAC2a, FEPD, OCI‐LY13.2) for 48 or 72 h and determined the number of dead cells via PI‐assay. The FACS analysis revealed for 3 out of 4 tested lines (KIJK, MAC2a and FEPD) significantly higher proportions of PI+ cells after IBL‐202 treatment compared to AZD1208+copanlisib treatment. OCI‐LY13.2 cells strongly responded to both, combi‐ and IBL‐202 treatment (Figure 5A). To exclude adverse side effects, human lymphocytes of healthy donors were treated in the same way as the mTCL cells. PI‐assay showed only slightly increased numbers of PI+ lymphocytes after IBL‐202 treatment when compared to DMSO‐treated controls that were not significant (data not shown).

GR_max_ values were calculated as described above. We plotted the GR_max_ values of IBL‐202 for the 17 mTCL lines in comparison to AZD1208, copanlisib, and the dual PI3K/mTOR inhibitor gedatolisib. As described above, GR_max_ values for AZD1208 mainly indicated cytostatic effects (GR_max_ > 0). For copanlisib, some of the tested lines showed GR_max_ values indicating a cytotoxic effect (GR_max_ < 0), whereas others showed a rather cytostatic or even no effect. Although most of the tested lines showed GR_max_ values < 0 on gedatolisib treatment, this was even surpassed by IBL‐202 with GR_max_ values being consistently < 0 (Figure 5B). Western Blot analysis verified the decrease of relevant phospho‐proteins that was comparable to AZD1208+copanlisib induced results (Figure S4).

**FIGURE 6:**
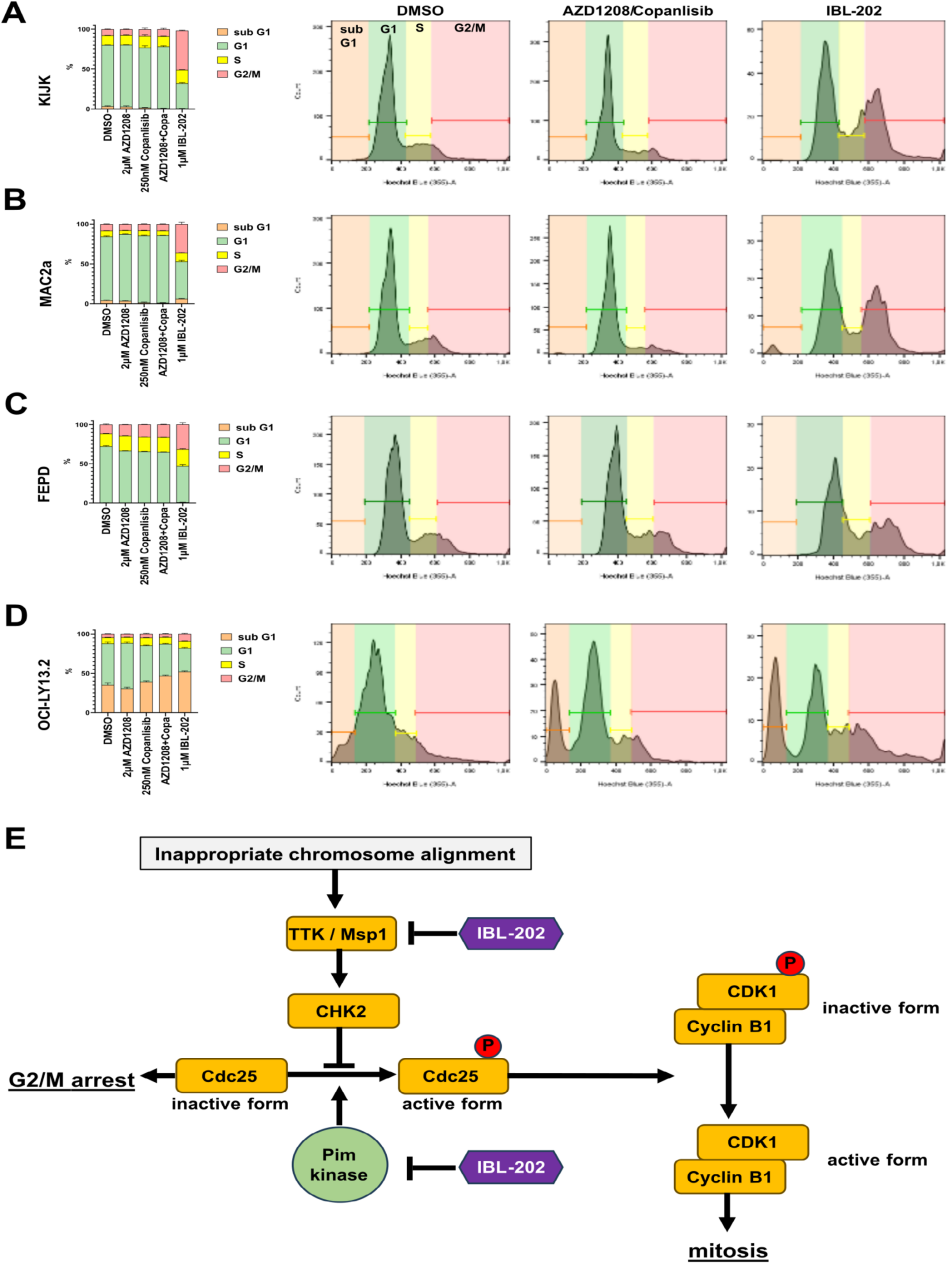
Cell cycle analysis. FACS‐based analysis of KIJK (A), MAC2a (B), FEPD (C), and OCI‐LY13.2 (D) cells treated for 72 h with either 2 μM AZD1208, 250 nM copanlisib, combination of both or 1 μM IBL‐202 was performed to analyze impact of the treatment on the cell cycle. DMSO‐treated cells were used as a control. Based on the Hoechst staining, cells were grouped into the cell cycle phase sub G1 (orange), G1 (green), S (yellow), and G2/M (red). For each cell line, the quantification is shown on the left as stacked bar diagram and representative FACS plots for DMSO, AZD1208+copanlisib or IBL‐202 groups are depicted on the right. The proposed underlying mechanism of the IBL‐202‐induced cell cycle arrest is graphically summarized in (E).

KinomeSCAN analysis was performed to detect interactions of IBL‐202 with relevant kinases. Among 455 tested kinases, interactions were apparent for 23 and mapped as a KinomeTree. Besides Pim kinases and different PI3K forms, IBL‐202 acted on threonine tyrosine kinase (TTK; also known as monopolar spindle 1, Msp1), c‐Jun N‐terminal kinases (JNK) 2 and 3, cyclin‐dependent kinase‐like 1 (CDKL1) and serum‐ and glucocorticoid‐regulated kinase (SGK) (Figure 5C). Especially, TTK has previously been described as a promising target for cancer treatment (reviewed in [20]). KinaseProfiler analysis further substantiated the IBL‐202‐induced TTK inhibition that was not observed for AZD1208 or copanlisib (IC_50_(nM) IBL‐202: 31.5, AZD1208: > 10 k, copanlisib: > 10 k). Cell cycle analysis of 4 mTCL lines was performed to determine whether IBL‐202‐induced TTK inhibition is acting on cell cycle checkpoints. We chose again KIJK, MAC2a, FEPD, and OCI‐LY13.2. Treatment with AZD1208 or copanlisib as well as with the combination of both for 72 h did not interfere with the cells' capability to run properly through the cell cycle phases. Thus, the distribution of the viable cells in the different cell cycle phases was nearly equal to DMSO‐controls. But in three out of four tested lines, 72 h of IBL‐202 treatment led to an increase in the proportion of cells that are stuck within the cell cycle phases G2/M. The IBL‐202‐induced G2/M arrest was most prominent in KIJK cells (Figure 6A) but was also apparent in MAC2a (Figure 6B) and FEPD (Figure 6C). OCI‐LY13.2 cells did not show a G2/M arrest (Figure 6D).

## 4 Discussion

Studies on the effects of Pim kinase inhibition on mTCL cells are sparse. Current knowledge mainly arises from one publication from 2014. In this study, researchers characterized the effects of siRNA‐mediated Pimknockdown and pharmacological inhibition with the pan‐Pim inhibitor ETP‐39010. The group described gross cytotoxicity of mTCL cells after pan‐Pim inhibition that is induced by accumulation of DNA damage [5]. Our study could show that the pan‐Pim inhibitor AZD1208 induced cytostatic effects but had very limited impact on the cell viability across a panel of 17 mTCL lines of different subgroups. However, additional inhibition of the PI3K/Akt pathway using the inhibitor copanlisib robustly induced cytotoxic effects. Similar results were described before, for example, for prostate cancer [21] or AML, where dual inhibition of Pim (AZD1208) and Akt (AZD5363) was able to induce cytotoxicity [22].

The interplay of Pim kinase‐ and PI3K/Akt‐pathways has been described before and can be traced back to the common downstream target mTOR (reviewed in [4]). The mTORC1 complex is a critical regulator of cellular translational machinery, with several downstream events. Both Akt and Pim can phosphorylate PRAS40 (an inhibitory subunit of the mTORC1 complex), which leads to the dissociation of p‐PRAS40 from the complex and subsequently an increased mTOR activity. The mTOR kinase phosphorylates 4EBP1 and p70S6 kinase, thereby regulating cap‐dependent translation [23, 24]. We used phosphorylation of S6, the substrate of the p70S6 kinase, as an indicator for the mTOR activity. AZD1208 showed reversible short‐term effects on the phosphorylation of S6 that led to decreased phosphorylation of S6 after 3 h, which returned to control levels until 24 h after inhibition start. This indicates that Pim kinases might be the driving force in mTOR activation, further substantiating their importance in the pathogenesis of mTCL. Hence, after shut‐down of the Pim kinase pathway, an upregulation of the PI3K/Akt axis neglects those effects, underpinning the need for combination therapy. Strikingly, in the PTEN‐deficient cell line OCI‐LY13.2 Pim inhibition showed only minor effects, but PI3K inhibition induced strong cytotoxicity [25]. The tumor suppressor PTEN acts as a negative regulator of the PI3K/Akt pathway, and LoF leads to stable activation of the PI3K/Akt pathway, constantly activating mTOR [26]. Therefore, Pim kinases might only have minor effects in cells with this particular mutation.

Evasion of intrinsic apoptosis is one of the main roles of Pim kinases in cancer cells. Among the Pim targets, for example, Bcl2 agonist of cell death (BAD) and apoptosis signaling kinase 1 (ASK1) act directly on apoptosis pathways. BAD phosphorylation at Ser112 can be induced by all three Pim isoforms. Phosphorylation blocks binding to BCL‐xL, and p‐BAD then binds to 14‐3‐3 that shuttles p‐BAD from the mitochondrial membrane to the cytosol, thereby preventing apoptosis ([27], reviewed in [28]). BH3 profiling showed that AZD1208 is able to prime BCL‐xL addicted cells for apoptosis. However, dependencies on the different factors regulating mitochondrial apoptosis are variable among different mTCL lines. Still, AZD1208+copanlisib treatment shows an overall ability to enhance BH3 peptide‐induced cytochrome C release, further underpinning the potency of this combination therapy.

The dual inhibitor of Pim and PI3K, IBL‐202, has proven efficacy against hematological cancers before. Pre‐clinical studies on multiple myeloma (MM) and chronic lymphocytic leukemia (CLL) revealed cytotoxic effects, especially under microenvironmental conditions leading to enhanced Pim expression [29, 30, 31]. One challenging factor in mTCL treatment is the heterogeneity of this entity, making it hard to find drugs that target the different subtypes. IBL‐202 showed cytotoxic effects on all tested mTCL lines, across different subtypes. Importantly, IBL‐202 showed high efficacy, even exceeding dual inhibition with the clinically applied drugs AZD1208 and copanlisib. It is known that Pim kinases act on the proliferation of cancer cells by interfering with the cell cycle checkpoints, by phosphorylating cell division cycle 25 (CDC25) family members that are important regulators of the G1/S (CDC25A, [32]) and G2/M (CDC25C, [33]) checkpoints. In healthy cells, p53 and the checkpoint kinases CHK1 and CHK2 inhibit the activity of CDC25C when DNA damage occurs and thereby arrest the cells in the G2/M phase. They do so by preventing the phosphorylation of CDC25C, leading to degradation of the molecule in the cytoplasm, preventing the activation of the cyclin B1/CDK1 complex [34]. By phosphorylating CDC25C irrespective of DNA damage, Pim kinases induce uncontrolled proliferation of cancer cells [33, 35]. Furthermore, KinomeSCAN shows the inhibitory function of IBL‐202 on TTK. High TTK activity is known as a predictor for unfavorable disease outcomes in different cancers including MM (reviewed in [17, 33]). It has been shown that genetic or siRNA‐mediated TTK depletion makes cancer cells susceptible to X‐ray induced G2/M arrest in vitro, as the kinase regulates CHK2 sensing improper chromosome alignment during anaphase [36, 37]. Upon cell cycle analysis, we detected that IBL‐202, but not AZD1208+copanlisib, was able to arrest cells at the G2/M checkpoint. We propose this to be caused by IBL‐202‐induced TTK inhibition (Figure 6E); still, further studies are needed to gain a deeper understanding of the impact of TTK in mTCL.

In summary, our study underscores the relevance of Pim kinases and the PI3K signaling pathway as therapeutic targets in mTCL and highlights dual inhibition with innovative compounds such as IBL‐202 as a novel therapeutic strategy in mTCL.

## Author Contributions

M.L., M.H., and R.K. designed and performed research, analyzed data, and wrote the manuscript. M.L., M.H., L.R., K.M., N.K., N.S., G.N., L.T., and R.K. designed and performed research and analyzed data. M.O., S.M.‐G., C.B.‐A., and D.C. analyzed data.

## Ethics Statement

The authors have nothing to report.

## Consent

The authors have nothing to report.

## Conflicts of Interest

Michal O'Neill and Darren Cunningham: employment at Inflection Biosciences. All other authors declare no competing financial interests.

## Supporting information


Figure S1.

Figure S2.

Figure S3.

Figure S4.

**Data S1.** Supporting Information.

## Data Availability

Data and materials supporting the results or analyses presented in this paper are available upon reasonable request.
