# Supplementary figures and images for: Dual Targeting of Pim and PI3 Kinases in Mature T‐Cell Lymphoma
Source: Eur J Haematol. 2025 Mar 31;115(1):82–95. doi: 10.1111/ejh.14420 (PMC12134715; doi:10.1111/ejh.14420)

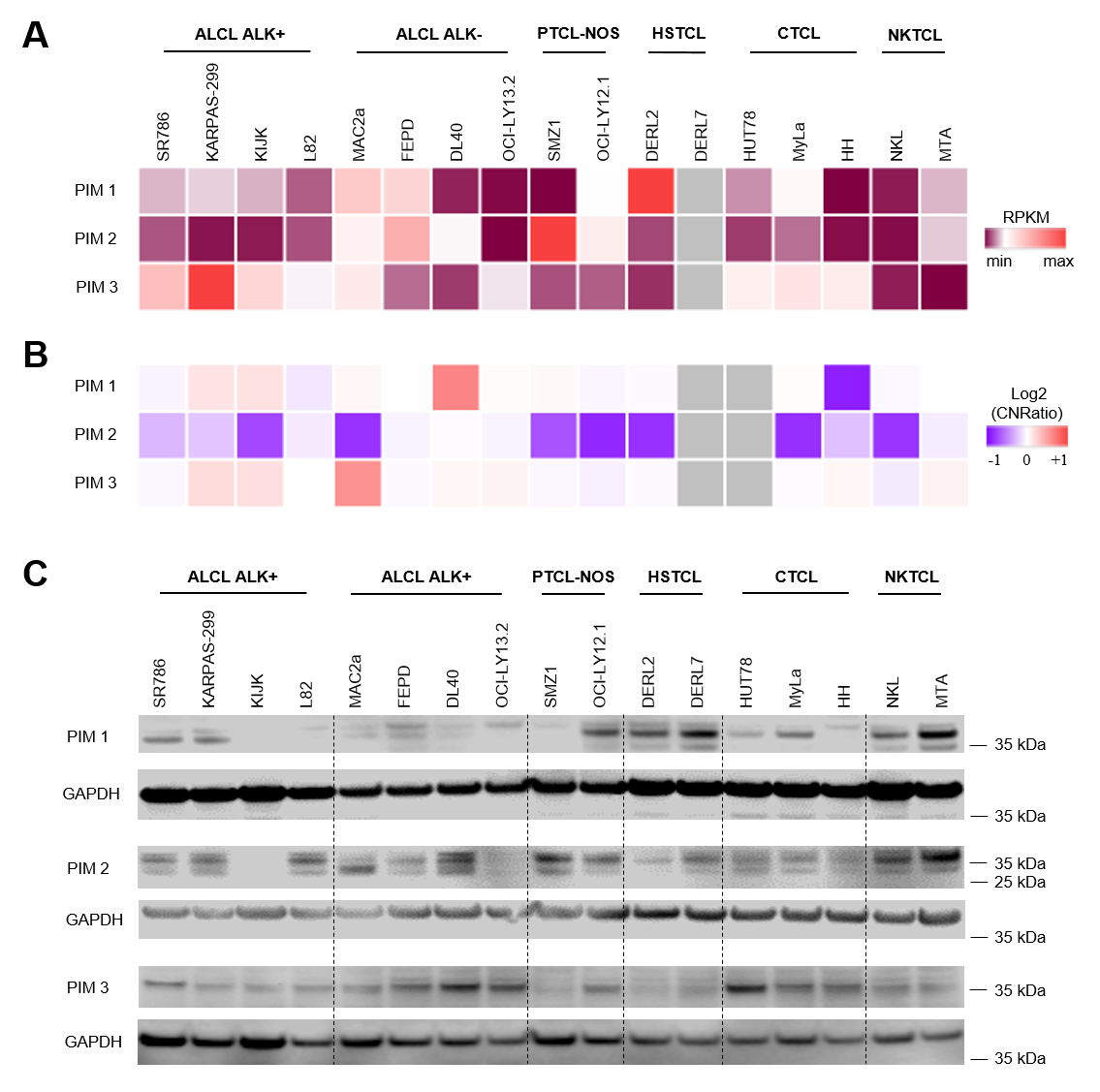

Supplement: Supplementary file 1 — Figure S1. [file EJH-115-82-s001.png]

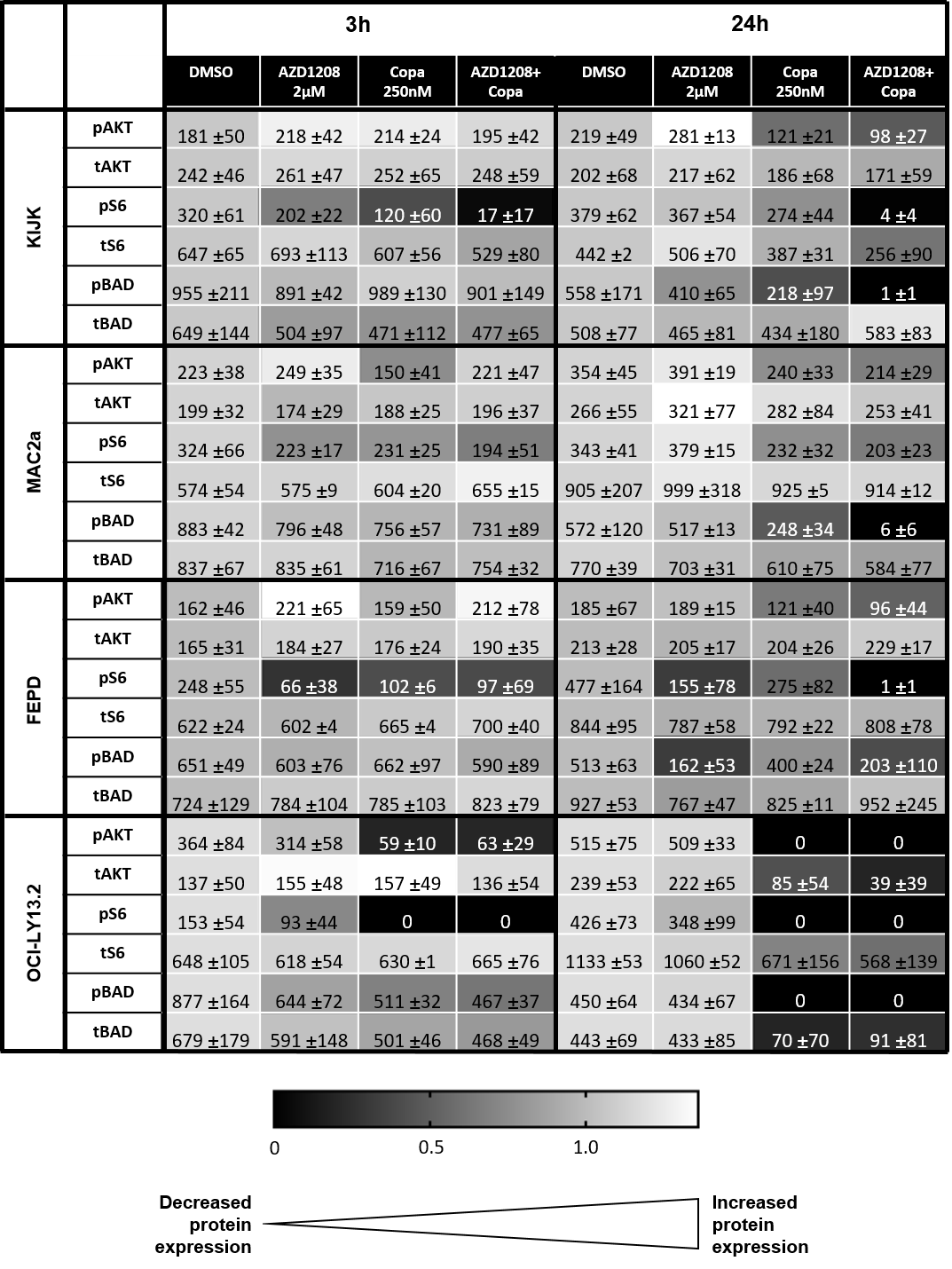

Supplement: Supplementary file 2 — Figure S2. [file EJH-115-82-s004.png]

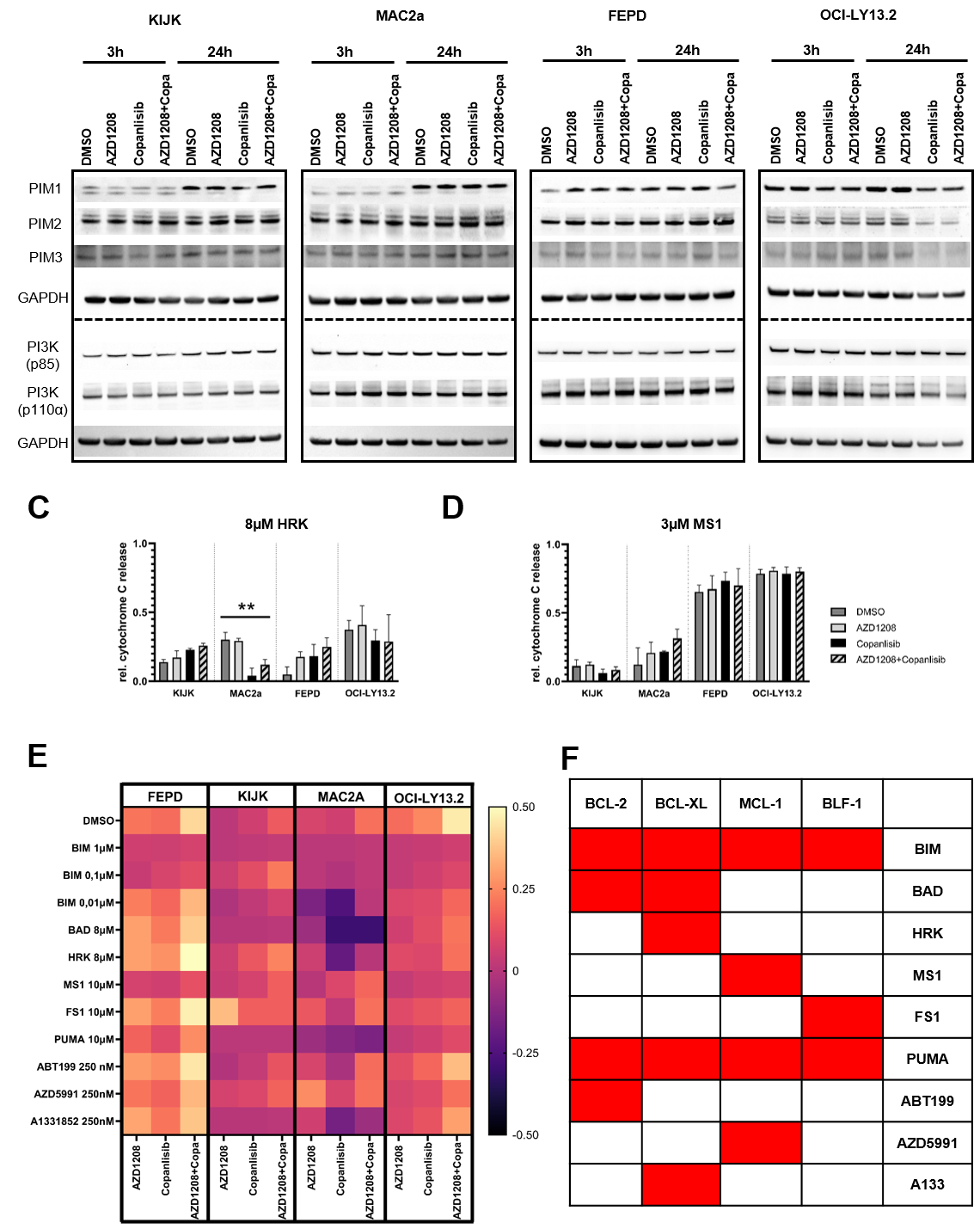

Supplement: Supplementary file 3 — Figure S3. [file EJH-115-82-s003.png]

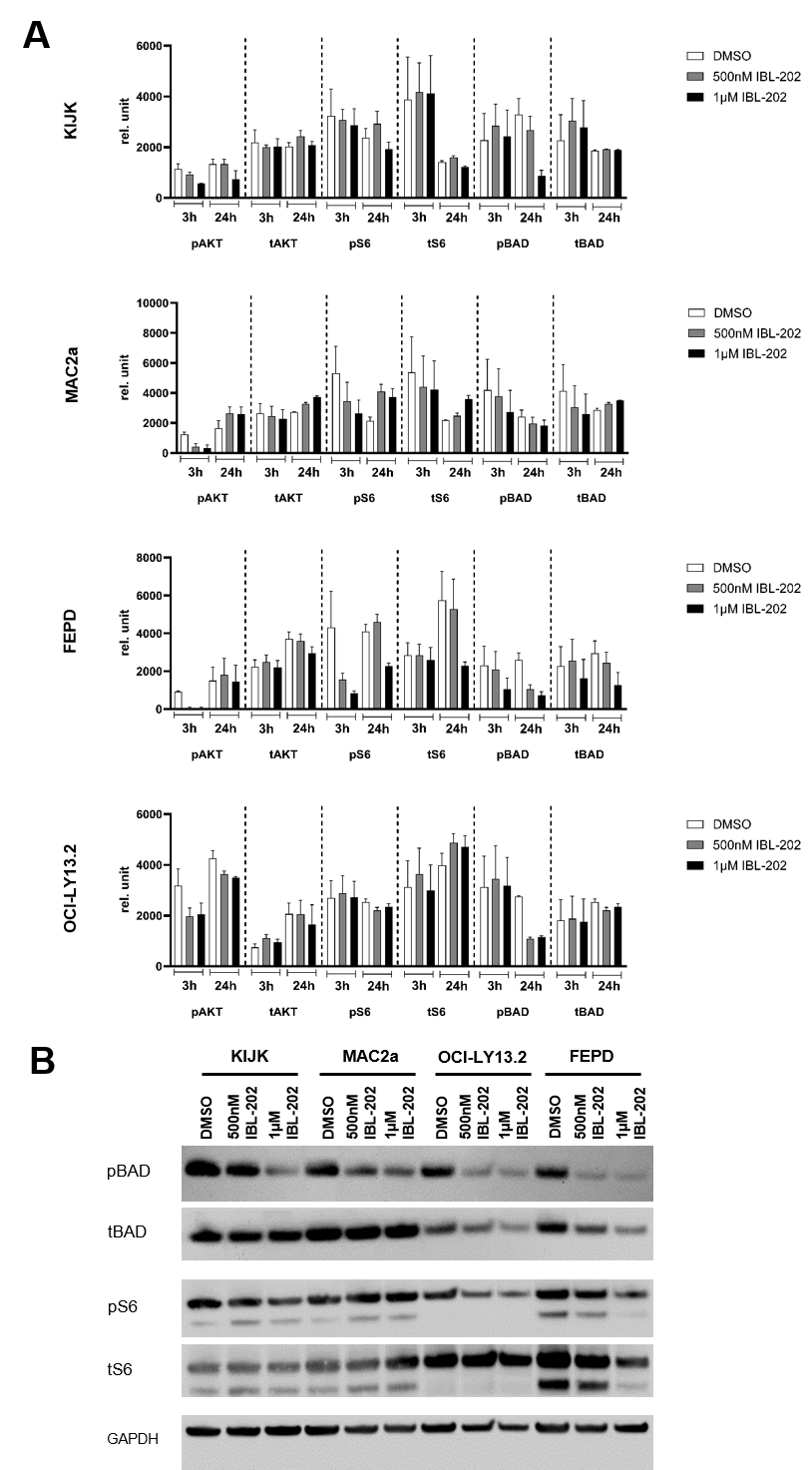

Supplement: Supplementary file 4 — Figure S4. [file EJH-115-82-s002.png]
