# Supplementary material for: Dual Targeting of Pim and PI3 Kinases in Mature T‐Cell Lymphoma
Source: Eur J Haematol. 2025 Mar 31;115(1):82–95. doi: 10.1111/ejh.14420 (PMC12134715; doi:10.1111/ejh.14420)
Supplement: Supplementary file 5 — Data S1. Supporting Information. [file EJH-115-82-s005.docx]

**Supplementary table 1.** T cell lymphoma lines.

| **Cell line** | **Subtype** | **Supplier (Cat. No.)** | **Culture conditions** |
| --- | --- | --- | --- |
| KARPAS-299 | ALCL ALK+ | Sigma Aldrich (06072604-1VL) | 20% FBS |
| KIJK | ALCL ALK+ | DSMZ (ACC 695) | 20% FBS |
| L82 | ALCL ALK+ | DSMZ (ACC 597) | 10% FBS |
| SR786 | ALCL ALK+ | DSMZ (ACC 369) | 20% FBS |
| DL-40 | ALCL ALK- | JCRB (JCRB1337) | 20% FBS |
| FEPD | ALCL ALK- | kindly provided by Leandro Cerchietti† | 10% FBS |
| MAC-2a | ALCL ALK- | kindly provided by Leandro Cerchietti† | 20% FBS |
| HH | CTCL | kindly provided by Anthony Letai§ | 10% FBS |
| HUT-78 | CTCL | ATCC (TIB-161) | 10% FBS |
| MyLa | CTCL | Sigma Aldrich (95051033-1VL) | 20% FBS |
| OCI-LY12.1 | HSTL | kindly provided by Leandro Cerchietti† | 10% FBS |
| DERL-2 | HSTL | DSMZ (ACC 531) | 20% FBS + 100 U/ml IL-2 |
| DERL-7 | HSTL | DSMZ (ACC 524) | 20% FBS + 100 U/ml IL-2 |
| MTA | NKTCL | JCRB (IFO50513) | 20% FBS |
| NKL | NKTCL | Kindly provided by Jerome Ritz§ | 20% FBS + 100 U/ml IL-2 |
| OCI-LY13.2 | PTCL-NOS | kindly provided by Leandro Cerchietti† | 20% FBS |
| SMZ-1 | PTCL-NOS | kindly provided by Hitoshi Ohno‡ | 20% FBS |

ALCL: Anaplastic large cell lymphoma, ALK±: with or without translocation of the anaplastic lymphoma kinase gene, CTCL: Cutaneous T-cell lymphoma; HSTL: Hepatosplenic T-cell lympoma; NKTCL: Natrural killer/T-cell lymphoma; PTCL-NOS: Peripheral T-cell lymphoma not otherwise specified.

† Weill Cornell Medicine, New York, NY, USA; ‡ Tenri Medical Institute, Tenri, Japan; § Dana Faber Cancer Instiute, Boston, MA, USA

RPMI 1640 medium (Cat. No. AC-LM-0058, Anprotec, Bruckberg, GER) supplemented with 10 or 20% FBS (Cat. No. AC-SM-0143, Anprotec, Bruckberg, GER) as specified in table S1, and 1% penicillin-streptomycin (Cat.No. P06-07100, PAN-Biotech, Aidenbach, GER) in a humidified incubator at 37 °C and 5% CO_2_.

**Supplementary table 2.** Half-maximal inhibitory concentration (IC_50_).

| **Cell line** | **Subtype** | **IC_50_ [µM]** | **Cell line** | **Subtype** | **IC_50_ [µM]** |
| --- | --- | --- | --- | --- | --- |
| KARPAS-299 | ALCL ALK+ | 1.5 x 10^-3^ | DL-40 | ALCL ALK- | 4.2 10^-2^ |
| KIJK | ALCL ALK+ | n.d. | FEPD | ALCL ALK- | 0.7 |
| L82 | ALCL ALK+ | 1.1 | MAC-2a | ALCL ALK- | 3.7 x 10^-3^ |
| SR786 | ALCL ALK+ | n.d. | OCI-LY13.2 | ALCL ALK- | 3.2 x 10^-3^ |
| HH | CTCL | 0.3 | DERL-2 | HSTL | 3.3 x 10^-2^ |
| HUT-78 | CTCL | 2.4 x 10-2 | DERL-7 | HSTL | 1.7 x 10^-2^ |
| MyLa | CTCL | 1.5 x 10^-3^ | MTA | NKTCL | 0.8 |
| OCI-LY12.1 | PTCL-NOS | 0.2 | NKL | NKTCL | 1.0 x 10^-2^ |
| SMZ-1 | PTCL-NOS | 9.1 x 10^-2^ |  |  |  |

ALCL: Anaplastic large cell lymphoma, ALK±: with or without translocation of the anaplastic lymphoma kinase gene, CTCL: Cutaneous T-cell lymphoma; HSTL: Hepatosplenic T-cell lympoma; NKTCL: Natural killer/T-cell lymphoma; PTCL-NOS: Peripheral T-cell lymphoma not otherwise specified; n.d.: not determinable.

**Supplementary figure 1**

**Analysis of the Pim-expression profile of mTCL cells lines.** (A) the expression levels of mRNA encoding for Pim-1, -2 or -3 were compared and plotted in a matrix format with reads per kilobase million (RPKM) Pim-mRNA ranging from burgundy red (low levels) to scarlet (high levels). (B) the appearance of copy number variations (CNVs) in the Pim-genes was plotted in a matrix format with Log2 values depicted. The Log2 values were ranging from -1 (one-copy loss, violet) over +0.57 (one-copy gain, light red) up to +1 (two-copy gain, scarlet). Log2 value of 0 indicates no CNV (white). Grey boxes indicate that no data was available. (C) the protein levels of Pim kinases and the house-keeper gene GAPDH were analyzed using western blot analysis. Representative pictures are shown for the 17 tested cell lines.

**Supplementary figure 2**

**Quantification of Western Blot experiments.** Western blot results were quantified using FIJI software. Results are depicted as relative values normalized to the house-keeping gene GAPDH. Results are given as mean ± SEM.

**Supplementary figure 3**

**Further analysis to decipher mechanisms of synergy.** Western blot analysis of protein lysates from KIJK, MAC2a, FEPD and OCI-LY13.2 cells after 3 or 24h pre-treatment with 2µM AZD1208, 250nM copanlisib or combination of both was performed analyzing Pim1, 2, 3 as well as different PI3 Kinase subunits (PI3K p85, PI3K p110α). Dynamic BH3 profiling was performed testing changes in Bcl-2 dependency of KIJK, MAC2a, FEPD and OCI-LY13.2 cells after 72h pre-treatment with 2µM AZD1208, 250nM copanlisib or combination of both. Control cells were treated with DMSO only. Values were normalized for each line to DMSO treated controls (DMSO group was set as 0). (C) depicts relative cytochrome C release in the presence of 8µM HRK and (D) in the presence of 3 µM of MS1 peptide. Results of three independently performed experiments are depicted as mean ± SEM. One-way ANOVA was performed with α=0.05 and * p<0.05, ** p<0.01 and ***p<0.001. (E) shows the delta-priming value (priming of the treated cells – priming of control cells) as a heat map. How the different peptides and BH3 mimetics act on the BCL-2, BCL-xL and MCL-1 pathway is depicted in (F).

**Supplementary figure 4**

**Analysis of relevant phospho-protein levels under IBL-202.** Western blot analysis of phosphorylated Akt (phos-Akt), total Akt, phosphorylated S6 (phos-S6), total S6, phosphorylated BAD (phos-BAD), total BAD as well as of the house-keeping gene GAPDH was performed on KIJK, MAC2a, FEPD and OCI-LY13.2 cells. Pre-treatment was performed for 3 or 24h with 500nM or 1µM IBL-202. Control cells were treated with DMSO only (n=1). (A) is the quantification of the different proteins, that is depicted as bar graphs. (B) shows representative pictures for pBAD/tBAD, pS6/tS6 and GAPDH after 24h treatment.

**Supplementary information on Western Blot analysis**

Protein isolation was done following standard protocols, using RIPA buffer (Cell Signaling #9806) containing PhosSTOP^TM^ (Roche #4906845001) and cOmplete mini protease inhibitor cocktail (Roche #11836153001). Proteins were stored at -80°C. For immunoblotting protein concentration was estimated using the Pierce^TM^ BCA Protein Assay (Thermo Fisher #23225). 25µg protein were diluted with NuPAGE^TM^ LDS Sample Buffer (4x) (Thermo Fisher), loaded on a gradient gel (NuPAGE^TM^ 4-12% Bis-Tris Gel, Thermo Fisher) and separated in an electrophoresis chamber. After wet blot transfer to a nitrocellulose membrane, membranes were blocked in 5% BSA in TBS-T for 90min at RT and subsequently primary antibody was added. Primary antibodies were diluted in block buffer (mk rb anti-GAPDH, 1:2000 (Proteintech #60004-1-ig); mk rb anti-Pim-1 clone D8D74, 1:1000 (Cell Signaling #54523); mk rb anti-Pim-2 clone D1D2, 1:1000 (Cell Signaling #4730); mk rb anti-Pim-3 clone D17C9, 1:1000 (Cell Signaling #4165); mk rb anti-phospho-Akt (Ser473) clone D9E, 1:1000 (Cell Signaling #4060); mk rb anti-phospho-S6 Ribosomal Protein (Ser235/236), 1:1000 in TBS-T instead of block buffer (Cell Signaling #4858); mk rb anti-phospho-Bad (Ser136) clone D25H8, 1:1000 (Cell Signaling #4366); mk ms anti-Bad clone C-7, 1:1000 (santa cruz #sc-8044); pk rb anti-PI3 Kinase p85, 1:1000 (Cell Signaling #4292); pk rb anti-PI3 Kinase p110α clone C73F8, 1:1000 (Cell Signaling #4249) and incubated over night at 4°C. Secondary antibodies were diluted in TBS-T (horse anti-mouse IgG, 1:2000, Cell Signaling #7076 or ms anti-rabbit IgG, 1:5000, santa cruz sc-2357) and incubated for 90min at RT. Finally, detection was reached by adding Crescendo Western Blot Substrate (Millipore WBLUR0500) or Signal Fire ECL Elite Substrate (Cell Signaling #12757).

**Supplementary information on AlamarBlue^TM^ cell viability assay**

Therefore, mTCL cells were seeded into 384-well microplates in normal culture media (~125’000 cells per well, 50µl total volume). Either the drug / drug combination in different concentrations or DMSO as a control were added to each cell line. According to manufacturer’s instructions, 5µl of alamarBlue^TM^ were added after 72h and incubated at 37°C for 4h. Baseline values were obtained by making an additional measurement at 0h. As alamarBlue^TM^ contains an indicator for redox-reactions, one can now measure the cell viability by photometric analysis of the emitted light (excitation λ=544nm, emission λ=590nm, SpectraMax M3, Molecular Devices). Experiments were performed in triplicates.

**Supplementary information on BH3 profiling**

Briefly, 384-well microplates were pre-coated with 16µM BAD in mitochondrial buffer (MEB: 150 mM mannitol, 10 mM HEPES-KOH pH7.5, 150 mM KCl, 1 mM EGTA, 0.1% BSA, 5 nM Succinate). The antibiotic peptide alamethicin was used as a positive control and DMSO was used as negative control. For membrane permeabilization 0.002% digitonin were added to MEB buffer. Then, cells were washed and resuspended in mitochondrial buffer. 15’000 cells/well in MEB buffer were added to the pre-coated wells with 1:1 v/v, diluting compounds to the final concentration (1µM BIM, 0.1µM BIM, 0.01µM BIM, 8µM BAD, 8µM HRK, 10µM MS1, 10µM FS1, 10µM PUMA, 250nM ABT199, 250nM AZD5991, 250nM A1331852), and incubated for 60 min at 25°C. After the incubation step, cells were fixed adding 4% PFA for 10 min and neutralization buffer (1.7 M Tris-Base; 1.25 M Glycin, pH 9.1) for an additional 5 min in a 1:1 ratio.

Afterwards, cells were stained with an anti-Cytochrome C antibody (Clone 6H2.B4, 1:400, BioLegend #612301) in staining buffer (1% Triton X-100, 0.1g/ml Albumine Fraction V, in PBS) over night at 4°C. Hoechst was added in a 1:40 dilution for nuclear stain.

**Supplementary information on PI3, mTOR and Pim kinase assay**

*1. Protein PI3 Kinase assay*

The PI3K-α kinase activity was measured by using the commercial ADP-GLo^TM^ kinase assay available from Promega (#33-016), which is a homogeneous assay to measure the accumulation of ADP, a universal product of kinase activity. The enzymes, PI3K (p110 α) was purchased from Cama Biosciences (#07CBS-0402A). The assay was done following the manufacturer’s recommendations, Envision (Perkin Elmer). The net luminescence signals were calculated by the subtraction of background luminescence values. Net luminescence was normalized against the control activity included (100 % PI3 kinase activity, without compound). These values were plotted against the inhibitor concentration and were fit to a sigmoid dose-response curve by using the Graphad software.

The kinase activity of PI3K isoforms was measured by using the commercial PI3-kinase (h) HTRF™ assay available from Millipore, following the manufacturer recommendations. PI3Kα (p110α/p85α) and PI3Kδ (p110δ/p85α) were used at 100 pM; PI3Kβ (p110β/p85α) and PI3Kγ isoforms (p110γ) at 500 pM and 4 nM respectively. ATP concentration was 50 times KMATP: 200 μM for PI3Kα and PI3Kδ, 250 μM for PI3Kβ and 100 μM for PI3Kγ. PIP2 was held at 10 μM. Values were normalized against the control activity included for each enzyme (i.e, 100% PI3K activity, without compound). These values were plotted against the inhibitor concentration and were fitted to a sigmoidal dose-response (variable slope) curve by using GraphPad Software. The obtained IC50 were converted to Kiapp according to Cheng-Prusoff equation for competitive inhibitors (Cheng, Y.; Prussoff, W.H. Biochem. Pharmacol. 1973, 22, 3099).

*2. Protein mTOR Kinase assay*

mTOR (FRAP1), LanthaScreen™ Tb-anti-p4EBP1 (phosphor-threonine 46) and GFP-4E BP1 were purchased from Invitrogen. Reaction conditions used were those recommended by the manufacturer. Values given are averages of two independent experiments performed in duplicate.

*3. Protein PIM Kinase assays*

The PIM kinase activities were measured in our laboratories by using the commercial ADP Hunter™ Plus assay (DiscoveRx Ref. #33-016), a homogeneous assay measuring ADP accumulation, as a universal product of kinase activity. PIM-1 and PIM-2 proteins were obtained by purification as it is described by Martinez-Gonzalez et. al. PIM-1 and PIM-3 was acquired from Millipore (Cat#14-738). The assay was done following general manufacturer recommendations and adapting protein and substrates concentrations to optimal conditions. Kinase buffer was 15 mM HEPES, pH 7.4, 20 mM NaCl, 1 mM EGTA, 0.02% Tween-20, 10 mM MgCl2 and 0.1 mg/mL LBGG (bovine γ-globulin). All PIM kinases assays were done at 100 µM PIM tide (ARKRRRHPSGPPTA), as peptide substrate, and 100 µM ATP. Protein concentration was 50, 200 and 350 pg/µl for PIM-1, 2 and 3, respectively. In order to calculate the IC50 of the described compounds, serial 1:5 dilutions were prepared and the reaction started by addition of ATP. Incubation was done for 1 h at 25 ºC. Reagents A and B (DiscoveRx) were sequentially added to the wells and plates were incubated for 30 min at 37 ºC. Fluorescence counts were read in a Victor instrument (Perkin Elmer) with the recommended settings (544 and 580 nm as excitation and emission wavelengths, respectively). Values were plot against inhibitor concentration and fit to a sigmoid dose–response curve with the GraphPad software.

**Synthetic Scheme for the synthesis of IBL-202**

a) LDA, THF, -78 ºC, 1 h; then I2, -78 ºC, 1h; b) 5-amino-6-methoxypyridin-3-yl)boronic acid pinacol esther; PdCl_2_(Ph_3_P)_2_; Na_2_CO_3_ aq, dioxane, 100 ºC, 1 h. c) 5-(BOC-aminomethyl) thiophene-2-boronic acid; PdCl_2_(Ph_3_P)_2_; Na_2_CO_3_ aq, dioxane, 100 ºC. d) methyl 3-(chlorosulfonyl)benzoate, pyridine, 0 ºC, 1h. e) lithium hydroxide monohydrate, dioxane: H_2_O (2:1), 1 h, rt. f) TFA-DCM, rt, 1 h. g) HATU, HOAt, DIPEA, DMF, rt.

**Experimental Procedures**

**7-Chloro-2-iodo-3-methyl-thieno[3,2-b]pyridine (2).** LDA (1.8 M in tetrahydrofuran/heptane/ethylbenzene, 2.88 ml, 5.188 mmol) was added to a mixture of 7-chloro-3-methylthieno[3,2-b]pyridine **1** (794 mg, 4.323 mmol) in THF (28.70 ml) at -78 ºC. The mixture was stirred at -78ºC for 1 hour, then a solution of I_2_ (1.37 g, 5.404 mmol) in THF (4.0 ml) was added to the anion slowly. The reaction mixture was stirred at -78 ºC for 1 hour, when the reaction was completed. Then EtOAc was added to the mixture at -78 ºC followed by the addition of an aqueous saturated solution of Na_2_S_2_O_3_. Aqueous layer was extracted with EtOAc (x3). The combined organic layers were washed with brine, dried (Na_2_SO_4_), filtered and concentrated. The residue was triturated from CH_3_CN (x3) to give the desired product **2** as a pale yellow solid (1.20 g, 3.883 mmol, 90% yield). ^1^H NMR (300 MHz, DMSO) δ 8.69 (d, *J* = 5.2 Hz, 1H), 7.63 (d, *J* = 5.1 Hz, 1H), 2.45 (s, 3H). LCMS (ESI): Rt = 4.94 min, m/z = 309.9/311.9 [M+ H]^+^; 333.0/331.0 9 [M+ Na]^+^.

**5-(7-Chloro-3-methyl-thieno[3,2-b]pyridin-2-yl)-2-methoxy-pyridin-3-ylamine (3).** The reaction was carried out in two batches of 696 mg each one. In a sealed tube charged with compound **2** (696 mg, 2.248 mmol) in 1,4-dioxane (17.60 ml), (5-amino-6-methoxypyridin-3-yl)boronic acid pinacol esther (675 mg, 2.698 mmol), Na_2_CO_3_ 2M (5.87 ml) and dichlorobis(triphenylphosphine)palladium(II) (160 mg, 0.225 mmol) was heated at 100 ºC for 1 h. The reaction mixtures of both batches were concentrated and the crude was purified by Biotage Flash Chromatography (40M silica cartridge) using cyclohexane-EtOAc gradient from 0% - 30% to give compound **3** pure (1.11 g, 3.633 mmol, 81 % yield). ^1^H NMR (300 MHz, DMSO) δ 8.68 (d, *J* = 5.1 Hz, 1H), 7.66 (d, *J* = 2.2 Hz, 1H), 7.60 (d, *J* = 5.1 Hz, 1H), 7.16 (d, *J* = 2.2 Hz, 1H), 5.28 (s, 2H. NH_2_), 3.94 (s, 3H), 3.32 (s, 3H). LCMS (ESI): Rt = 4.65 min, m/z = 306.0/308.0 [M+ H]^+^.

**{5-[2-(5-Amino-6-methoxy-pyridin-3-yl)-3-methyl-thieno[3,2-b]pyridin-7-yl]-thiophen-2-ylmethyl}-carbamic acid tert-butyl ester (4).** The reaction was carried out in 2 batches. In a sealed tube charged with compound **3** (597 mg, 1.952 mmol) in dioxane (15.25 ml), 5-(BOC-aminomethyl)thiophene-2-boronic acid (614 mg, 2.343 mmol), Na_2_CO_3_ 2M (5.10 ml) and dichlorobis(triphenylphosphine)palladium (II) (138 mg, 0.195 mmol) was heated at 100 ºC for 1 h. Excess of boronic acid, palladium was added heating the mixture till completion of the reaction. The reaction mixtures of both batches were concentrated and the crude was purified Biotage (40M silica cartridge) using cyclohexane-EtOAc, gradient from 0% - 50% to give compound **4** (2 g) contaminated with Ph_3_PO but it was used in next reaction step as such. LCMS (ESI): Rt = 4.78 min, m/z = 483.1 [M+ H]^+^.

**3-(5-{7-[5-(tert-Butoxycarbonylamino-methyl)-thiophen-2-yl]-3-methyl-thieno[3,2-b]pyridin-2-yl}-2-methoxy-pyridin-3-ylsulfamoyl)-benzoic acid methyl ester (5).** The reaction was carried out in two bathes of 659 mg and 865 mg of compound **4**. To a solution of **4** (659 mg, 1.365 mmol) in Pyridine (9.60 ml) at 0 ºC was added methyl 3-(chlorosulfonyl)benzoate (384 mg, 1.639 mmol). The reaction mixture was stirred for 1 h at 0 ºC. Then MeOH was added, and stirred for 30 min at 0 ºC. The solvent was evaporated and the crude from both batches was purified by Biotage Flash Chromatography (25 M silica cartridge) using Cyclohexane-EtOAc gradient from 0% - 50% to give compound **5** (810 mg, 1.190 mmol, 38% yield). LCMS (ESI): Rt = 4.98 min, m/z = 681.2 [M+ H]^+^.

**3-(5-{7-[5-(tert-Butoxycarbonylamino-methyl)-thiophen-2-yl]-3-methyl-thieno[3,2-b]pyridin-2-yl}-2-methoxy-pyridin-3-ylsulfamoyl)-benzoic acid (6).** To a solution of **5** (810 mg, 1.190 mmol) in 1,4-dioxane (12 ml) and water (5.80 ml) was added to room temperature lithium hydroxide monohydrate (509 mg, 11.897 mmol). The reaction mixture was stirred for 1 h. The reaction mixture was concentrated and the crude was purified by Biotage Flash Chromatography (25 M silica cartridge) using Cyclohexane-EtOAc gradient from 0% - 100% and then with EtOAc-MeOH gradient from 5% - 30% to give compound **6** (598 mg, 0.897 mmol, 75% yield). LCMS (ESI): Rt = 4.75 min, m/z = 667.2 [M+ H]^+^.

**3-{5-[7-(5-Aminomethyl-thiophen-2-yl)-3-methyl-thieno[3,2-b]pyridin-2-yl]-2-methoxy-pyridin-3-ylsulfamoyl}-benzoic acid trifluoroacetate salt (7).** To a solution of 6 (598 mg, 0.897 mmol) in DCM (7.10 ml) at 0 ºC was added dropwise TFA (10.50 ml). The reaction mixture was stirred for 1 h at rt. Then the reaction mixture was concentrated and coevaporated with toluene (x3) affording a yellow solid crude, compound **7** (924 mg), which was used in the next step without further purification. LCMS (ESI): Rt = 3.26 min, m/z = 567.1 [M+ H]^+^.

**IBL-202.** The reaction was carried two batches of 462 mg each. The crude compound **7** (462 mg, 0.449 mmol theoric) was dissolved in DMF (18.50 ml) and DIPEA (0.60 ml, 3.393 mmol) was added. The mixture was slowly added (via syringe pump; 2 ml/h) to a solution of HATU (348 mg, 0.897 mmol) and HOAt (0.5 M in DMF, 1.80 ml, 0.897 mmol) in DMF (70.50 ml). After the addition, the reaction mixture was stirred for 14 h. Then it was evaporated and the crude from both batches was purified by Biotage Flash Chromatography (25 M silica gel) using Cyclohexane-EtOAc gradient from 0% - 100% to give desired compound, which was washed with MeOH (x3) and CH_3_CN (x3) to give pure compound **IBL-202** (147 mg, 0.268 mmol, 30 % over two steps). ^1^H NMR (700 MHz, DMSO) δ 10.74 (s, 1H), 9.87 (t, *J* = 6.0 Hz, 1H), 8.73 (d, *J* = 5.0 Hz, 1H), 8.61 (s, 1H), 8.33 (d, *J* = 1.6 Hz, 1H), 8.30 (d, *J* = 7.8 Hz, 1H), 8.18 (d, *J* = 7.6 Hz, 1H), 7.86 (t, *J* = 7.8 Hz, 1H), 7.75 (d, *J* = 5.0 Hz, 1H), 7.69 (d, *J* = 3.7 Hz, 1H), 7.62 (d, *J* = 1.9 Hz, 1H), 7.22 (d, *J* = 3.6 Hz, 1H), 4.80 (sbroad, 2H), 4.05 (s, 3H), 2.63 (s, 3H). LCMS (ESI): Rt = 4.88 min, m/z = 548.9 [M+ H]^+^.
